# Combined Effects of Clime, Vegetation, Human-Related Land Use and Livestock on the Distribution of the Three Indigenous Species of Gazelle in Eritrea

**DOI:** 10.3390/ani13091490

**Published:** 2023-04-27

**Authors:** Futsum Hagos, Tecle Yemane, Kamal M. Ibrahim, Marco Mangiacotti, Roberto Sacchi

**Affiliations:** 1 Hamelmalo Agricultural College, Keren P.O. Box 397, Eritrea; 2 Ministry of Land Water and Environment, Asmara P.O. Box 5713, Eritrea; 3 School of Biological Sciences, Southern Illinois University Carbondale, Carbondale, IL 62901, USA; 4 Department of Earth and Environmental Sciences, University of Pavia, 27100 Pavia, Italy

**Keywords:** Dorcas gazelle, Soemmerring’s gazelle, Heuglin’s gazelle, Eritrea, conservation and management, habitat selection, human disturbance, protected areas

## Abstract

**Simple Summary:**

*Nanger soemmerringii, Gazella dorcas* and *Eudorcas tilonura* are the three species of gazelle indigenous to Eritrea. Their status, distribution and habitat selection are poorly studied or unknown. This study fills this knowledge gap by providing the first data on environmental preferences and threats arising from human activities (agriculture and livestock) for the Eritrean populations of the three species. The distribution of the three species is mainly driven by climate and human-related disturbance rather than habitat features. Species tend to avoid agricultural areas and, particularly, areas with high density of livestock. To ensure the persistence of the three gazelle species in the country, it is urgent and decisive to establish targeted protected areas, as well as taking actions to reduce the impact of competition with livestock.

**Abstract:**

The status and habitat selection of the three species of gazelle indigenous to Eritrea, i.e., *Nanger soemmerringii, Gazella dorcas* and *Eudorcas tilonura,* are not well known. In this study, we analyzed the present distribution of the three species in the country in order to identify preferred habitats and assess the effect of human disturbance (land use for agricultural purposes and livestock) on species occurrence. These data represent baseline information for evidence-based strategies for conservation of the three species in Eritrea. Presence/absence data of the three species in each of the 67 administrative subregions (Sub Zoba) composing the country were collected using direct (field surveys) and indirect methods (questionnaires). For each sampling unit, we collected fifteen environmental variables, of which three are associated with climatic features, eight with vegetation structure and four with human disturbance (human-related land use and livestock). The occurrence probability of each species was modeled through Generalized Linear Models (GLM). The analyses showed that Dorcas gazelle occurred more frequently in warmer conditions and in a wide range of natural vegetation types. Heuglin’s gazelle occurred in warmer regions with higher seasonality in both temperature and precipitation with a preference for closed woody and open grassland areas. In the case of Soemmerring’s gazelle, the GLM with climatic variables predicted a preference for warmer conditions but with lower seasonality of temperature and precipitation. The species also seemed to prefer arid and semi-arid open vegetation. Human disturbance is the variable with the strongest, negative, effect on the species occurrence. Indeed, the occurrence probability of each species decreased with increasing livestock density and agricultural land use. Most of these gazelle occurred in unprotected areas, thus the human-related activities are undoubtedly the most important threat for the three species of gazelle in Eritrea. Therefore, the establishment of protected areas that preserve the potential optimal habitats for gazelle and reduce the impact of livestock ranching are essential to ensure a future for these gazelle in Eritrea.

## 1. Introduction

Ungulate species inhabiting arid regions of Northern Africa are seriously threatened, and several species risk extinction [1]. Among Antelopes, 31% of extant species are formally regarded as threatened, and 9% as nearly threatened [2]. Furthermore, the population trend is decreasing for 64% of assessed species and stable for 33% [2]. The primary reasons for species decline are certainly intensified hunting, habitat loss or deterioration as well as competition with domestic livestock [2,3,4].

Eritrea hosts three species of autochthonous gazelle, i.e., Soemmerring’s gazelle, *Nanger soemmerringii* (Cretzschmar, 1828), Dorcas gazelle, *Gazella dorcas* (Linnaeus, 1758) and Heuglin’s gazelle, *Eudorcas tilonura*, Heuglin, 1863.

**Figure 1:**
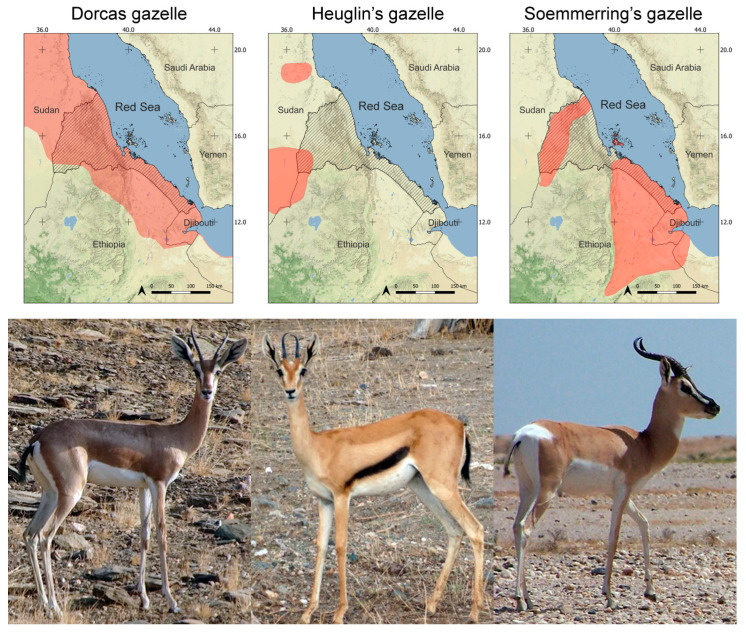
Historical distribution of the three gazelle species (light red layers) occurring in Eritrea (dashed area) according to IUCN [10,11,12] data. From left to right: Dorcas gazelle (*Gazella dorcas*), Heuglin’s gazelle (*Eudorcas tilonura*) and Soemmerring’s gazelle (*Nanger soemmerringii*).

Soemmerring’s gazelle is endemic to the Horn of Africa (Eritrea, Ethiopia, Djibouti, Somalia and southeastern Sudan, Figure 1). Its population in these regions is declining and the total population in its range is estimated at less than 10,000 [1]. In Awash National Park, Ethiopia, where it is legally protected, populations of Soemmerring’s gazelle have declined more than other antelopes [5]. The Sudanese populations have probably been extirpated [6]. In Eritrea, the species occurs in the coastal area, Dahlak Kebir Island and in restricted inland areas. Notably, Soemmerring’s gazelle is particularly abundant on Dahlak Kebir, probably due to the lack of terrestrial predators and the benign attitude of the local inhabitants, whose culture and ethical norms respect the wildlife [7,8]. However, the knowledge about the general status of the species in the country is scanty, even though Eritrea is currently a stronghold for the conservation of the Soemmerring’s gazelle [9].

Dorcas gazelle previously had the most extensive distribution of any African gazelle, but recent study revealed that the species is no longer present in several areas of its former range [13,14]. It was considered extinct from the mid-1970s in Senegal [15], where it was reintroduced in 2007. According to the IUCN [1], the decline of the species is estimated to be more than 30% over a period of about 15 years (up to April 2016), and fewer than 25% of those remaining at that time lived in protected areas. Furthermore, in the Sahara region, the Dorcas gazelle no longer inhabited 86% of its former range [16]. Consequently, the Dorcas gazelle is categorized as a globally vulnerable species [1]. In Eritrea (Figure 1), the species has been reported from the coastal area, in the south-western and northern parts of the country [17,18]. However, accurate information on the distribution and status of the species does not currently exist.

Heuglin’s gazelle, also known as the Eritrean gazelle, is endemic to the Horn of Africa, specifically to western Eritrea, NE Ethiopia and SE Sudan (Figure 1). In the past, it was considered a subspecies of the red-fronted gazelle (*E. rufifrons*) or conspecific with Thomson’s gazelle (*E. thomsonii*) and the Mongalla gazelle (*E. albonotata*) [17]. Other authors consider Heuglin’s gazelle an independent species [19,20], and this taxonomic status has been provisionally followed on the IUCN Red List [21]. According to the IUCN Red List, the species is classified as endangered. Across its range, the main threats are hunting, competition with domestic livestock and habitat degradation [20]. As stated by the IUCN SSC antelope specialist group, the population of the species might have fallen by 20% in roughly nine years since 2008 [1], and, currently, the estimated global population of the species ranges between 2500 and 3500 [22].

The status and distribution of the species in Eritrea are not well known. For more than 80 years, no Heuglin’s gazelle had been reported in the country; researchers lost sight of the species in the 1930s, and it has not been recognized in Eritrea since then. There have been no confirmed sightings of the species by professionals until 2019, when a small group of animals was observed and photographed in the region of Gash Barka (Zoba), between subregions of Dige and Gonge [23].

These three gazelle species used to be widely distributed in Eritrea until the first half of the 20th century [22], particularly Dorcas and Soemmerring’s gazelle (Figure 1). However, habitat degradation, chronic armed conflicts, drought and limited conservation actions have led to a serious decline in their abundance as well as shrinkage of their ranges. During the 30 years of war for independence (1961–1991), wild animals were taken as food by soldiers and many species declined, dispersed or became locally extinct [24]. After the independence of the country (1993), the Eritrean government has adopted a series of policies and practices, including the banning of hunting (1995 [24]), the establishment of protected areas and a national environmental management plan that emphasized community engagement, which have eventually allowed wildlife recovery [25]. As a result, the status of wildlife in the country is improving [26], and the revival of the three species of gazelle is now evident [23].

However, gazelle species are still under threat of human pressure, which may compromise their future existence. In order to prevent this scenario and help plan conservation strategies, it is decisive to update the knowledge about their distribution, the potential ecological drivers and the impact of human activities and livestock. This can help determine protection areas of high priority and highlight essential habitat management [27]. Furthermore, grazers can have critical effects on plant communities across a wide range of ecosystems or impact on vegetation and soils in different environments, as well as regulating plant abundance in aquatic ecosystems [28,29]. Therefore, understanding the ecological preferences and distribution of gazelle at a large geographic scale may also be of relevant importance for ecosystem management.

Ecological preferences of the three gazelle species in Eritrea are either not known or poorly studied. Similarly, the impact of human-related activities on species occurrence has never been studied for the Eritrean populations. With this in mind, this study attempted to (i) fill the gap of knowledge on the distribution of the three gazelle species throughout the country, (ii) identify the main environmental drivers of their current distribution and (iii) assess the possible effects of livestock and land use by human activities (namely, urbanization and agriculture) on gazelle. The findings of this study are pivotal to inform an evidence-based strategy for conservation of the three species in Eritrea.

## 2. Materials and Methods

### 2.1. Geographical Setting of the Country and Sample Size

Eritrea is situated in the Horn of Africa and lies north of the equator between 12°22′ N and 18°02′ N latitude, and 36°26′ E and 43°13′ E longitude. It has an area of 124,300 square kilometers with a mainland and island coastline of more than 3300 km [24]. The country is bordered by Sudan to the north and west, by Djibouti to the southeast and by Ethiopia to the south (Figure 1). The country is roughly divided into three physiographic regions, namely, the central highlands, the midlands and the lowlands [22]. Three quarters of the country (more than 74%) falls in the arid or semi-desert zones [30]. The climate is arid to semi-arid with high temperatures year-round. Mean annual daily temperature is approximately 30 to 35 °C, but during the hottest months, the maximum temperature can exceed 50 °C [25]. Rainfall is erratic, and both its amount and season varies depending on the region: in coastal areas, the rain season occurs between October and March, whereas inland, it extends between March–April and September–August [31]. The mean annual rainfall is usually less than 200 mm and 600 mm in the coastal and inland zones, respectively. The three species of gazelle occur, even in sympatry, in three sizeable, geographically separated areas: the western lowlands (Gash Barka Region), the Red Sea coastal areas (Southern and Northern Red Sea Region) and the Danakil desert (Southern Red Sea Region). Only one species (*Nanger soemmerringii*) also occupies the Dahlak Kebir island in the Red Sea.

Eritrea is divided into 6 main administrative regions (Zoba) and 67 subregions (Sub-Zoba). We used these subregions as sampling units to report the presence/absence of each species, the environmental variables used to define species’ ecological preferences and the anthropogenic impact. For the purpose of this study, the 13 subregions of the central Zoba (Asmara) were excluded from the analyses due to its irrelevance in terms of extension (less than 0.2%), thus analyses referred to a sample of 54 subregions.

### 2.2. Species Occurrence

To examine the occurrence of the three gazelle species within the selected subregion, different methods were applied, including direct (field surveys) and indirect observations (questionnaire) [32]. Field data were gathered along nine targeted surveys in the three geographically separated areas (inland, coastal area and island) during dry and wet seasons in 2021 and 2022. For each gazelle group or individual sighted, data on location, group size and habitat type were recorded. As much as possible, the observed species were photographed using a standard HD digital and video camera. To appraise the occurrence of the three gazelle species in the entire country, data questionnaires were also circulated within the historical species range (Supplementary Materials). As [33] pointed out, questionnaires can be precise and powerful tools for collecting an enormous amount of carefully focused information from a large number of people. This task was accomplished through collaborators found in respective Zobas (regions) selected among scouts and/or experts of plant protection from the Ministry of Agriculture, who frequently travel to the field to assess crop and rangeland condition. In addition, environmental experts from Bisha Mining Share Company and Colluli Potash Mining were also involved. Data coming from surveys and questionnaires were combined and used to classify each of the 54 subregions included in the sample as suitable or not suitable depending on whether the species has been recorded at least once or not, respectively.

### 2.3. Environmental and Human Disturbance Variables

To relate gazelle occurrence to environmental conditions and anthropogenic pressures, for each subregion, we collected variables describing climate, vegetation and human disturbance.

To characterize climate, we used the 19 bioclimatic variables from the WorldClim data series (www.worldclim.org/version 1.4) [34], cut out on the study area. The map resolution was ~1 km, and the unsigned Pearson correlation among variables was on average 0.47 (range: 0.01–0.99). The climatic principal component analysis (CPCA) produced three climatic principal components (CPCs) that explained 89.0% of the full variance and were used for modeling. Based on the CPCs’ loads (Table S1) we were able to interpret each of those three CPCs, as it is summarized in Table 1. For each subregion, we picked the mean value of the CPC scores.

The vegetation data were sourced from the Africover Project, available at the United Nations website (www.un-spider.org). According to the FAO Land Cover Classification System (LCCS), the shape file of the Eritrea maps 69 different land cover categories, grouped into seven main classes. Natural and semi-natural vegetation potentially affecting the environmental preferences of the three gazelle species are included in four of them: natural and semi-natural terrestrial vegetation (LCCS class: A12 with 26 land cover categories), natural and semi-natural aquatic vegetation (LCCS class: A24 with 4 land cover categories), bare areas (LCCS class: B16 with 10 land cover categories) and inland natural waterbodies (LCCS class: B28 with 5 land cover categories). The three other classes, i.e., cultivated terrestrial areas and managed lands, artificial surfaces and associated areas, and artificial waterbodies were not considered for this analysis. The vegetation categories from the four selected classes were grouped in 8 natural and semi-natural vegetation categories (VEG, Table 1), which were used to assess habitat preferences by the three target species. For each subregion, the coverage of each VEG class was computed and related to the subregions. The unsigned Pearson correlation among VEGs was on average 0.18 (range 0.01–0.72), so we did not perform any data reduction, and we used all VEGs as independent variables in statistical analyses.

Human disturbance was assessed by two sets of variables associated with livestock and land use. The number of livestock (cattle, sheep, goats, donkeys and camels) for each subregion was obtained from the Ministry of Agriculture of the state of Eritrea 2021 report (unpublished). Values were converted into density (n/ha) to make it comparable among subregions. The unsigned Pearson correlation among the five groups of livestock was on average 0.46 (range: 0.02–0.69), so we summarized them using a PCA. The analyses produced two components that explained 81.1% of the full variance and were used for modeling. Based on the PCs’ loads (Table S2), we were able to interpret each of these two PCs (hereafter “livestock PCs”, LPCs), as summarized in Table 1. Furthermore, we still used LCCS to assess the impact of human activities on natural environments, and specifically, we used the built-up areas of any nature (LCCS class: B15, Artificial surfaces and associated areas, hereafter URB) and crops (LCCS class A11, Cultivated terrestrial areas and managed lands, hereafter CLT). The Pearson correlation was 0.30 and variables were standardized before being added to the model as independent predictors.

### 2.4. Statistical Analyses

To analyze the effect of climate on species occurrence, we modeled the probability to observe a species in a subregion through a Generalized Linear Model (GLM) with binomial error distribution, where CPC1, CPC2 and CPC3 entered the model as predictors. One different and independent model was performed for each species. Then, we used the same approach to analyze the effect of natural vegetation on the probability of observing a given species. In this case, all 8 VEGs entered the model as linear predictors. Finally, a third GLM was used to assess the effect of human disturbance with LPC1, LPC2 and standardized URB and CLT as predictors.

In order to assess if the negative effects of livestock on the species occurrence were due to direct interference (i.e., inter-specific interactions not mediated by the environment, such as active exclusion though aggressive interaction) or indirect interaction mediated by another factor (e.g., for resources such as food or water due to the common occurrence in the same habitats), the last GLM was run a second time after controlling for the effect of VEG on disturbance variables. This was carried out by running four linear models, one for each human disturbance variable, with the eight VEG variables entered as predictors. The residuals of these four models were included as predictors in the GLMs accounting for the effects of human disturbance on species occurrence instead of the four original variables. In summary, significant effects in these GLMs could be interpreted as a sign of direct negative interactions between gazelle and livestock.

Models were fitted in a Bayesian analytical framework available through the R (v. 4.2.1) package ‘brms’ [35], which uses the samplers implemented in STAN. Uninformative normal priors (μ = 0 and σ = 100) were used for model’s coefficients. Three chains were run using randomly selected initial values for each parameter within a reasonable interval, and conventional convergence criteria were checked. The number of iterations was selected for each run to obtain at least 10,000 valid values for each chain after convergence and thinning. Results from the posterior distribution are reported as the half sample mode (HSM, [36]) with 95% and 50% highest density intervals (HDI_95_ and HDI_50_, respectively [37]).

## 3. Results

**Figure 2:**
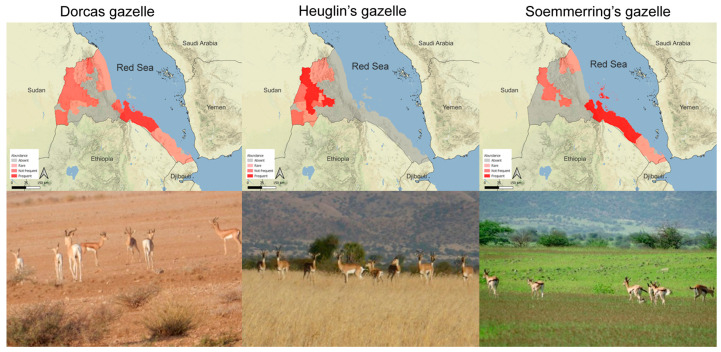
Current distribution of Dorcas, Heuglin’s and Soemmerring’s gazelle in Eritrea. Lower panels from left to right: Soemmerring’s and Dorcas gazelle occasionally seen feeding together (Buri peninsula); Dorcas and Heuglin’s gazelle observed to overlap using the same area alternatively in different seasons with Soemmerring’s gazelle; same habitat utilized alternatively by Heuglin’s and Soemmerring’s gazelle (Duluk, Gash Barka).

The most frequent and widely sighted species was the Dorcas gazelle, which was observed at least once in 18 sampling units (33%). This includes the following subregions: Monsura, Akurdet, Dige, Gogne, Mogolo, Kerkebet, Adobha, Afabet, Korora, Foro and all subregions found along the Red Sea coastline (Figure 2). The Eritrean gazelle was observed in 23% of sampling units, corresponding to the subregions of Monsura, Mogolo, Dige, Gogne, Forto-Sawa, Kerkebet, Golig and Adobh (Figure 2). Finally, Soemmerring’s gazelle shows the more restricted distribution, highly concentrated in coastal and island areas, mainly in the subregions Ghelaelo, Araeta and Dahlak Kebir Island (Figure 2). Each of the three species recurrent observations concentrated in more restricted areas (Table 2), corresponding to the two subregions of Ghelaelo and Araeta for the Dorcas gazelle (Figure 2); the five subregions of Kerkebet, Dige, Monsura, Gogne and Adobha for the Eritrean gazelle (Figure 2); and the coastline between Foro and Maekel Denkalia for the Soemmerring gazelle (Figure 2). In addition, Dahlak Kebir Island hosts an abundant population of Soemmerring’s gazelle. Finally, the Dorcas gazelle was repeatedly observed to occur in association with Soemmerring’s gazelle and occasionally with Eritrean gazelle in respective areas of their range (Figure 2, lower panels).

### 3.1. Climatic and Natural Vegetation Preference—Ecological Niche of Dorcas Gazelle

**Figure 3:**
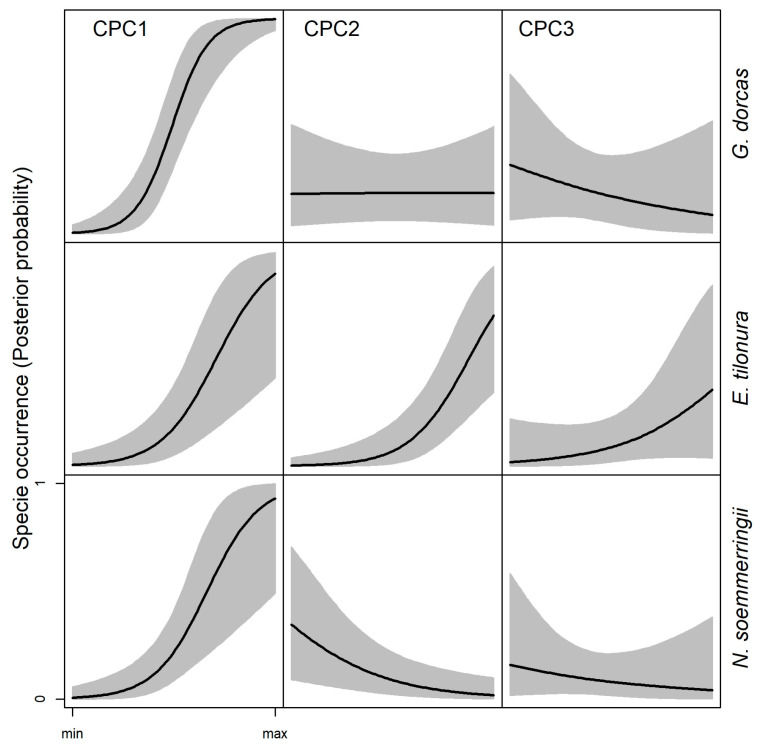
Bayesian model prediction for the probability of species occurrence in response to the climatic variables. The scale’s X-axis ranges from the minimum to the maximum of the variable; solid lines indicate HSM, and the gray areas represent HDI_95_.

**Figure 4:**
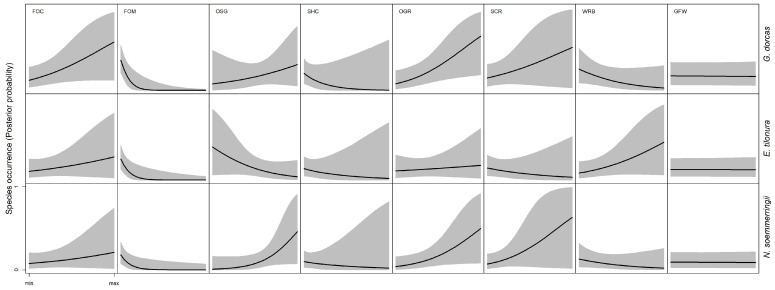
Bayesian model prediction for the probability of species occurrence in response to the climatic variables. Solid lines indicate HSM, and the gray areas represent HDI_95_.

The GLM for the climatic variables (Table 3) showed that the Dorcas gazelle occurred more frequently in warmer conditions (higher solar radiation, CPC1, P_β>0_ > 0.99), with reduced thermal seasonality (CPC3, P_β<0_ = 0.79, Figure 3), whereas no effect was observed for rainfall seasonality (CPC2, P_β>0_ = 0.50, Figure 3). According to the model for VEG variables (Table 3), the species occurred in a wide range of natural vegetation, including herbaceous, shrubs and woody areas, but with different trends depending on the vegetation structure. Indeed, the occurrence probability increased in closed woods (FOC, P_β>0_ = 0.96) but decreased in mixed woody areas (FOM, P_β>0_ < 0.001, Figure 4). The opposite trend appeared for shrubs. In this case, the model indicated a preference for open and sparse shrub grassland (OSG, P_β>0_ > 0.79) compared to closed ones (SHC, P_β>0_ = 0.10, Figure 4).

Accordingly, open grassland vegetation promoted the species’ occurrence (OGR, P_β>0_ = 0.99, Figure 4), as well as open scrublands on rocky and compact grounds (SCR, P_β>0_ = 0.83, Figure 4). Finally, riverbanks (WRB) had a slightly negative effect (P_β<0_ = 0.94), whereas grassland in swampy areas (GFW) did not show any relevant effect on species presence (P_β>0_ = 0.42, Figure 4).

### 3.2. Climatic and Natural Vegetation Preference—Ecological Niche of Heuglin’s Gazelle

The model for climatic variables (Table 2) predicted the Heuglin’s gazelle in Eritrea occurring in warmer regions (CPC1, P_β>0_ > 0.99), with higher seasonality in both precipitation (CPC2, P_β>0_ > 0.99) and temperature (CPC3, P_β>0_ = 0.92, Figure 3). The model with VEG variables (Table 2) indicated a preference by the species for closed woody areas (FOC, P_β>0_ = 0.79, Figure 4) and riverbanks (WRB, P_β>0_ = 0.92, Figure 4). On the other hand, mixed woods (FOM), open shrub grassland (OSG) and open scrubland (SCR) are generally avoided (P_β<0_ > 0.90, Figure 4).

### 3.3. Climatic and Natural Vegetation Preference—Ecological Niche of Soemmerring’s Gazelle

The GLM with climatic variables (Table 2) predicted for the Soemmerring’s gazelle, as for the other two species, a preference for warmer condition (CPC1, P_β>0_ > 0.99), but associated with reduced seasonality in both precipitation (CPC2, P_β<0_ > 0.99) and temperatures (CPC3, P_β<0_ = 0.74, Figure 3). According to the model for VEG variables (Table 2), the species occurs more frequently in arid and semi-arid open vegetation. Indeed, the model detected a clear preference for open shrub grassland (OSG), open grassland (OGR) and open scrubland on rocky ground (SCR, P_β>0_ > 0.89, Figure 4). Riverbanks (WRB) and mixed woods (FOM) were generally avoided by the species (P_β<0_ > 0.87, Figure 4).

### 3.4. Human Disturbance

**Figure 5:**
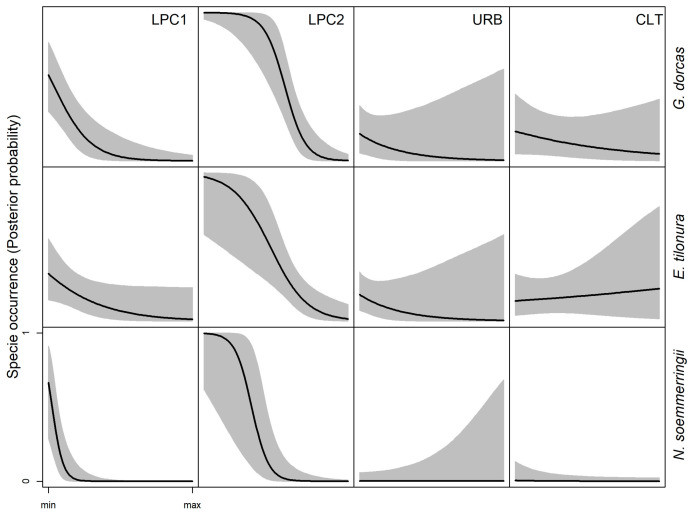
Bayesian model prediction for the probability of species occurrence in response to the human disturbance as estimated through livestock (LPC1 and LPC2) and human-related land cover (URB and CLT). Solid lines indicate HSM, and the gray areas represent HDI_95_.

The GLMs with human disturbance variables (Table 2) were consistent among the three species of gazelle in predicting a negative effect on species occurrence of both livestock and human-made environments (Figure 5). The probability of gazelle occurring decreased with increasing livestock density (LPC1, P_β<0_ > 0.97) and also with increasing prevalence of cattle, goats and camels with respect to sheep and donkeys (LPC2, P_β<0_ > 0.99). The effect of human-related land use was less intense compared to that of livestock, but it was still negative (Figure 5).

Models predicted a clear negative effect of the urban areas (URB, Figure 5) for Dorcas gazelle (P_β<0_ = 0.88) and Heuglin’s gazelle (P_β<0_ = 0.89) but not for Soemmerring’s gazelle (P_β<0_ = 0.46). The negative effects of cultivated areas (CLT, Figure 5) on species occurrence were detected for Dorcas gazelle (P_β<0_ = 0.83) and Soemmerring’s gazelle (P_β<0_ = 0.92) but not for Heuglin’s gazelle (P_β<0_ = 0.36).

**Figure 6:**
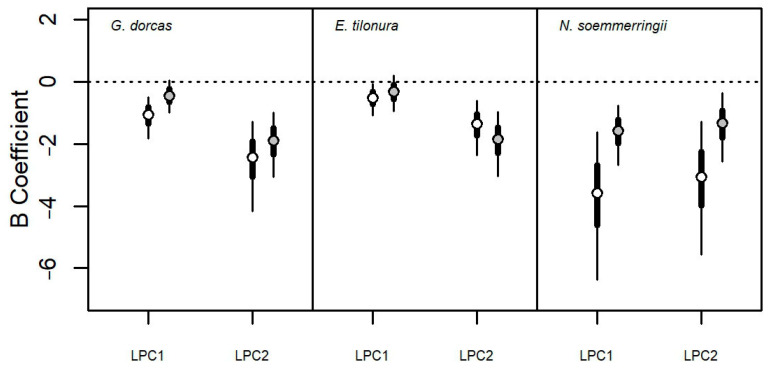
Comparison between the effects (posterior distribution of model β coefficients) of human disturbance on species occurrence estimated by Bayesian GLM with (gray dots) or without (white dots) the control for the effect of species ecological preferences (see Methods for details). Circles indicate HSM, and thick and thin lines represent HDI_50_ and HDI_95_, respectively.

When controlling for the effect of VEG variables, the negative effects of livestock density and composition on species occurrence were fully confirmed (P_β<0_ > 0.84 in all cases; Figure 6). The negative effect of livestock abundance on species occurrence was less intense in Dorcas and Soemmerring’s gazelle (P_βdiff<0_ > 0.90, Figure 6), but no difference occurred for the Heuglin’s gazelle (P_βdiff<0_ = 0.66, Figure 6). With regard to the effect of livestock composition, the difference between models followed the same pattern as for livestock abundance (Figure 6). The negative effect of livestock composition was less intense after controlling the effects of VEG variables for Dorcas (P_βdiff<0_ = 0.71) and Soemmerring’s (P_βdiff<0_ = 0.90) gazelle but slightly increased in Heuglin’s gazelle (P_βdiff<0_ = 0.27).

## 4. Discussion

The survey confirmed that the three species of gazelle still exist in wide parts of Eritrea, even though in most subregions, sightings of the species are either null or rare. *N. soemmerringii* gazelle are widely distributed in the Red Sea coastal area, Dahlak Kebir Island and the mainland (the southwest part of the country). The coastal area appears to have the highest abundance of both *N. soemmerringii* and *G. dorcas* compared to the inland population of the species. Interestingly, *N. soemmerringii*, *G. dorcas* and *E. tilonura* are sympatric inland, whereas in coastal areas, *N. soemmerringii* and *G. dorcas* were observed to coexist. On Dahlak Island, only *N. soemmerringii* occurs with viable population.

The observed geographic distribution of the three species may reflect the slight difference in their ecological niches, at least according to climatic and VEG models. Indeed, while they all prefer hotter regions, *E. tilonura* seems to associate with climate regimes with more seasonality than the other two species. On the other hand, *N*. *soemmerringii* seems more related to areas with less seasonality, especially in temperature. Considering VEG variables, *E. tilonura* is associated with wooded grassland, and it is frequently observed in riverine vegetation. These latter findings agree with those of the Ethiopian populations [38] from the Kafta Shiraro National Park (which shares a border with Eritrea), where Heuglin’s gazelle showed preference for exactly the same kind of habitat (i.e., wooded grassland). Contrary to the current study, the avoidance of both open and closed shrubs (SHC, SHO) was not detected in the Ethiopian populations and in previously reported data [20,38]. The reason for this variation could be due to the habitat encroachment by agriculturalist. It is common to see agricultural practice interspersed with open shrubland and or grassland habitats. Consequently, in this case, human disturbance rather than habitat unsuitability may be the reason for gazelle to move to other areas, leading models to predict an apparent avoidance of open areas. The preference by Heuglin’s gazelle for riverine vegetation is in agreement with what is known about the species [20], which is reported to be more water-dependent than other species of gazelle in the same region: riparian areas have more green vegetation throughout the year, and this could support gazelle with physiological water.

By contrast, the environmental niches of *N. soemmerringii* and *G. dorcas* are much more similar to one another than the niche of *E. tilonura*. The models revealed that the most preferable habitats identified were open woods or shrub grasslands followed by open grasslands (herbaceous), suggesting that these species are more adapted to arid and semi-desert habitats. Accordingly, the two species of gazelle are the only ones occurring in the Danakil Depression, which is one of the harshest parts of the world. The main difference in the vegetation structure of open wood/shrubs and grasslands between coastal regions and inland is that, in the former, a savannah structure with grassland dominated by *Aristida mutabilis* and *A. adcensionis* prevails, while, in the latter, a semi-arid vegetation, dominated by grass, e.g., *Pancium turgidum* and *Cynodon dactylon*, prevails. Therefore, it can be suggested that in Eritrea, the Heuglin’s gazelle is exclusively a savannah dweller, whereas the Dorcas and Soemmerring’s gazelle inhabit both savannah and open desert grassland.

With regard to the Dorcas gazelle, our results show that it tends to inhabit a wider range of habitats than Soemmerring’s gazelle. Indeed, the species has been frequently observed in open shrub grasslands, grasslands, scrublands, in arid to semi-desert areas, including the Danakil Depression. These findings are in line with reports for the populations in Tunisia [39], where the Dorcas gazelle avoids areas with agricultural development and did not select any particular habitat feature, occurring in grassland, shrubland and semi-desert. Furthermore, our findings also parallel data from the Sahara’s Grand Erg region in southern Tunisia [40], where the Dorcas gazelle is more affected by human disturbance than land use. Human activities force the species to avoid more humid and greener areas, in a way that such suitable habitats are perceived by gazelle as too dangerous and are avoided in favor of less suitable but safer habitats. On the other hand, our findings tend to dispute with the research carried out in Egypt’s Eastern Desert [41] concerning the effects of precipitation patterns. Indeed, the study on Egyptian Dorcas gazelle concluded that the species may not be able to withstand long periods of drought, especially because of spatially unpredictable precipitation. Our study showed that one of the most frequented areas by the species is in the harshest area of Eritrea, with very low precipitation. However, this does not necessarily mean that those habitats are the preferred ones, as we cannot exclude that constant observations in the Danakil Depression may be due to human avoidance. Nevertheless, this finding suggests that the Eritrean populations of Dorcas gazelle are well adapted to drought and desert environments and able to respond to long periods of drought through behavioral adaptations, such as movements over the territory or changes in daily activity and habitat preferences.

Among the three species, the Soemmerring’s gazelle is the one with the narrowest distribution and the highest presence in the Danakil Depression. Accordingly, the climatic and environmental GLMs revealed that the most preferred habitats of the species are open shrub grassland and open grassland. These results totally agree with a similar study performed in the Alledeghi Wildlife Reserve, Eastern Ethiopia [42], where Soemmerring’s gazelle shows preference for grassland habitat during the wet season and for bushland habitat during the dry season. However, the species in Eritrea has a strong tolerance to high temperatures, also due to behavioral and physiological adaptations. We repeatedly observed the species during the harshest season (July–August), when temperatures rise beyond 45 °C, escaping from heat stress by staying inside mangroves until the temperatures drop. Furthermore, the gazelle are able to maximize the efficiency of water conservation through concentrating urine and adjusting body temperature and evaporative cooling [43].

The adaptation of Soemmerring’s gazelle to drought could be a problem in terms of conservation when combined with the fact that the species is much more sedentary than the other gazelle. Indeed, sedentary animals are susceptible to drought due to their low mobility, which makes them eventually unable to escape the effects of drought conditions [43,44]. Therefore, Soemmerring’s gazelle are exposed to death from thermal shock when temperatures become particularly high, and this risk can increase because sedentary habits also expose individuals to shortage of food during the dry season. Actually, in the coastal area, when the temperature rises to 50 °C, the death of gazelle due to heat stress may be a common phenomenon (Scout Mohamed Ahmed, pers. comm.).

A second important result of this study is the negative effects of human activities on species occurrence, especially due to livestock. For every species, models systematically predict the probability of gazelle occurrence to decrease with increasing livestock density, and the effect was more pronounced for cattle, goats and camels compared to sheep and donkeys. The effect of human-related land use, notably agriculture, was less intense compared to that of livestock but still had a negative impact on the species occurrence, particularly for Dorcas gazelle.

Economic development is transforming the pattern of land use in savannah ecosystems, promoting a systematic shifting of uncultivated land towards an economically profitable land use, thus eroding habitats suitable for gazelle outside protected areas [45]. For example, the change from the nomadic to a sedentary lifestyle in southern Tunisia caused a privatization of lands and the creation of farmlands for cereals and olive crops [39]. Consequently, the habitats preferred by Dorcas gazelle were eroded, and species occurrence severely declined [39]. There is general support that livestock is among the main threat for gazelle, and more generally, antelope conservation (e.g., [12,34,40]). Negative effects might be both direct, through preventing access to the best foraging areas (e.g., high stocking density) [45,46], or indirect, through optimal habitat alteration by grazing (e.g., forage reserve depletion or shrub encroachment in open habitats) [45,46,47]. However, the effects of livestock are not always negative, and in some cases, cattle coexist with and even facilitate wild grazers [46]. Nevertheless, the avoidance of areas with high livestock density may be caused by human herders who accompany livestock. Higher human presence may also lead to higher human predation on gazelle for food or to reduce competition for livestock.

Despite the fact that the status of gazelle indigenous to Eritrea is probably improving, also thanks to several conservation actions [7,23], the species are facing both natural and human-induced threats. In this study, we found that interaction between livestock and gazelle in the grazing area is probably the main threat to the survival of gazelle in Eritrea. The problem is more pronounced in inland (Gash-Barka), where livestock reaches the highest density, particularly goats, which have similar feeding behavior to gazelle. In addition, the absence of protected areas for wildlife allows livestock to graze and browse freely, resulting in severe habitat degradation. In the absence of proper land use planning, the conservation status of the three species of gazelle is not expected to improve in the long term and will most likely render the fate of these gazelle in Eritrea uncertain. Therefore, in view of the existing magnitude of the threat that the gazelle face, it is highly important to think about how to best manage and conserve what remains: there is a dire need for appropriate land use strategies to ensure coexistence of gazelle and pastoralists.

## 5. Conclusions

Ecosystems in African savannahs can be better conserved if management is based on a clear understanding of the threats to wildlife dynamics and on their interaction with livestock. We showed that the distribution of the three species of gazelle indigenous to Eritrea is mainly driven by climatic and human-related disturbance rather than habitat features. Species tend to avoid agricultural areas and, particularly, areas with high livestock density. Our results suggest some recommendations for future planning of conservation actions in favor of the three species of gazelle. First, the gazelle’s preferred habitats need active protection; thus, there is a dire need for the establishment and operationalization of protected areas in the country. Second, specific protected areas should be established in those areas that are facing serious human–wildlife conflicts. Such types of protected area need to be preferably IUCN category VI (multiple use) type. The creation of a network of unfenced conservation areas in which livestock densities are persistently low and which are sufficiently large to act as ‘sources’ of individuals that are able to disperse is desirable. Some forms of subsidy could be used to support the income of ranching during dry years in order to promote the coexistence of gazelle with livestock [46]. Geographically, priority needs to be given to such areas that are facing serious threat from the expansion of agriculture and pastoralists. Further studies are also needed in order to evaluate population dynamics of the three gazelle species in the country.

## Figures and Tables

**Table 1:** List of variables used in the analyses of ecological preferences and human impact of the three target species of gazelle analyzed in this study. The third column reports the changes in the variables captured by PC scores (climatic and livestock variables) or the land cover uses in the FAO Land Cover Classification System (LCCS).

Variable	Description	Pattern of Variation/LCCS Codes
Climatic variables		
CPC1 (50.6%)	Solar radiation (bio01, bio05, bio06, bio08–bio11)	From low to high
CPC2 (27.7%)	Rainfall seasonality (bio02, bio07, bio15, bio16)	From low to high
CPC3 (10.7%)	Temperature seasonality (bio04, bio18)	From low to high
Natural and semi-natural vegetation (VEG)		
FOC	Closed woody vegetation	2WC
FOM	Mixed forest with shrubs	2TC128, 2TC328
OSG	Open shrub grassland	2TP28, 2TP68, 2TR6, 2SOJ67, 2SP6, 2SPJ6, 2SPM58, 2SV6, 2SVJ67, 2SR6, 2SR6//6ST1, 2SR6//6ST2
SHC	Closed shrubs	2SCJ
OGR	Open grassland	2HR(CP), 2HR(CP)8, 2HR, 2HR//6L, 2HR//6S, 2HR//6ST1
SCR	Open scrubland on rocky and stony ground	6L, 6S, 6R, 6G
WRB	Riverbanks	8WFN1
GFW	Close to very open grassland in swampy areas (fresh water)	4H(CP)F8, 4HCF
Human disturbance		
LPC1 (59.0%)	Livestock density	From low to high
LPC2 (22.1%)	Livestock composition (Prevalence of cattle, goats and camels vs. sheep and donkeys)	From low to high
URB	Urban areas	5A, 5P, 5U
CLT	Crops	HD4, HD57, HL57, HR4, HR57, ND57, NR57, TBED47PL, TBEL57V, SBE57V

**Table 2:** Occurrence of the three species of gazelle indigenous to Eritrea in the 55 subregions monitored in this study. Rare, not frequent and frequent correspond to hard to see, occasional and persistent observation respectively.

Species	Absent	Present		
*G. dorcas*	37 (67%)	18 (33%)		
		Rare	Not frequent	Frequent
		7 (11%)	9 (50%)	2 (39%)
*E. tilonura*	42 (76%)	13 (23%)		
		Rare	Not frequent	Frequent
		6 (46%)	3 (23%)	4 (31%)
*N. soemmerringii*	44 (80%)	11 (20%)		
		Rare	Not frequent	Frequent
		3 (27%)	3 (27%)	5 (46%)

**Table 3:** Posterior distributions of the species occurrence as estimated by Bayesian GLM. HSM and HDI_95_ estimates are shown. Human disturbance values refer to the model without control for ecological effects on species occurrence (see methods for details).

Variables	*Gazella dorcas*		*Eudorcas tilonura*		*Nanger soemmerringii*	
	β (HDI_95_)	P_β<0_	β (HDI_95_)		β (HDI_95_)	P_β<0_
Climatic variables						
CPC1 (solar radiation)	0.85 (0.47; 1.40)	<0.001	0.55 (0.22; 0.98)	<0.001	0.58 (0.24; 1.09)	<0.001
CPC2 (rainfall seasonality)	0.01 (−0.32; 0.33)	0.50	0.95 (0.43; 1.72)	<0.001	−0.47 (−0.90; −0.12)	>0.99
CPC3 (temperature seasonality)	−0.23 (−0.89; 0.32)	0.79	0.50 (−0.20; 1.3)	0.08	−0.22 (−0.98; 0.42)	0.74
Natural and semi-natural vegetation (VEG)						
FOC	35.3 (−4.24; 79.2)	0.04	18.4 (−26.9; 62.3)	0.21	18.0 (−30.1; 63.7)	0.22
FOM	−147.4 (−294.2; −38.8)	>0.99	−124.7 (−271.5; −19.4)	0.99	−112.7 (−262.5; −10.0)	0.99
OSG	1.94 (−2.74; 7.19)	0.21	−3.23 (−7.9; 1.26)	0.92	5.37 (−0.6; 13.0)	0.04
SHC	−70.4 (−227.1; 30.4)	0.90	−32.8 (−146.2; 43.0)	0.79	−25.9 (−166.4; 62.0)	0.69
OGR	5.53 (0.59; 11.1)	0.01	0.99 (−4.05; 6.1)	0.35	5.69 (−0.29; 12.7)	0.03
SCR	2.36 (−2.5; 7.53)	0.17	−2.06 (−7.95; 2.73)	0.80	4.1 (−2.44; 11.1)	0.11
WRB	−90.5 (−217.3; 24.3)	0.94	82.9 (−29.4; 196.1)	0.08	−71.0 (−202.1; 48.5)	0.87
GFW	−19.1 (−213.2; 176.1)	0.58	−8.31 (−199.2; 185.3)	0.53	−20.3 (−217.0; 172.3)	0.59
Human disturbance						
LPC1 (livestock density)	−1.07 (−2.03; −0.41)	>0.99	−0.51 (−1.21; 0.02)	0.97	−3.59 (−6.98; −1.34)	>0.99
LPC2 (livestock composition)	−2.45 (−4.57; −1.11)	>0.99	−1.36 (−2.58; −0.46)	>0.99	−3.08 (−6.16; −1.02)	>0.99
URB	−0.60 (−2.15; 0.33)	0.88	−0.61 (−2.02; 0.31)	0.89	0.04 (−1.09; 0.99)	0.46
CLT (crops)	−0.37 (−1.27; 0.37)	0.83	0.14 (−0.64; 0.87)	0.36	−0.90 (−2.84; 0.30)	0.92

## Data Availability

Not applicable.

## Supplementary Materials

The following supporting information can be downloaded at: https://www.mdpi.com/article/10.3390/ani13091490/s1, Questionnaire, Table S1, Table S2.
