# Supplementary material for: Combined Effects of Clime, Vegetation, Human-Related Land Use and Livestock on the Distribution of the Three Indigenous Species of Gazelle in Eritrea
Source: Animals (Basel). 2023 Apr 27;13(9):1490. doi: 10.3390/ani13091490 (PMC10177417; doi:10.3390/ani13091490)
Supplement: Supplementary file 1 [file animals-13-01490-s001.zip › animals-2331374-supplementary.pdf]

Supplementary Material

Data collection process on species occurrence

In addition to the direct method of data collection, to appraise the distribution and conservation status of the three gazelles in the entire country and obtain quantitative data on species occurrence, questionnaires (Appendix I) were circulated to collaborators available in the different regions and sub regions (Zoba). Most of them are wildlife scouts, experts from the Ministry of Agriculture who frequently travel to the field for assessing crop and rangeland conditions. Besides, environmental experts from Bisha Mining Share Company and Colluli Potash Mining were also involved. In order to maintain consistency among areas, all selected collaborators were briefly trained to data collection methodologies and species identification.

Supplementary Materials S1. Questionnaires circulated to collaborators.

Zoba ______________________________ Sub Zoba ____________________

Specific location __________________________ Date____________________

Name of the data collector ______________________________________

Observed species	Number	Northing	Easting	Habitat	Date	Occurrence*

[…]

* *Occurrence articulated as:*

Rare: few occasionally species detection (species observation is hardly ever),

Not frequent: for sporadic species detection, (observation is not common) and

Frequent: stands for common (regular) species**.**

Table S1. PC loads for the worldclim variables

Worldclim	PC1	PC2	PC3
bio_01	0.301	0.115	-0.151
bio_02	-0.088	0.414	-0.090
bio_03	-0.243	0.122	-0.345
bio_04	0.220	-0.114	0.419
bio_05	0.284	0.186	-0.102
bio_06	0.304	-0.006	-0.211
bio_07	0.068	0.392	0.148
bio_08	0.283	0.125	0.103
bio_09	0.293	-0.041	-0.271
bio_10	0.307	0.103	-0.090
bio_11	0.275	0.155	-0.258
bio_12	-0.276	0.112	-0.238
bio_13	-0.236	0.284	-0.082
bio_14	-0.174	-0.222	-0.137
bio_15	-0.077	0.425	0.098
bio_16	-0.220	0.295	-0.153
bio_17	-0.145	-0.312	-0.192
bio_18	-0.209	0.040	0.351
bio_19	-0.009	-0.195	-0.403

Table S2. PC loads for the livestock variables

variable	PC1	PC2
CATTLES	0.485	0.096
SHEEP	0.452	-0.341
GOATS	0.516	0.189
DONKEY	0.493	-0.367
CAMELS	0.228	0.839
